# Multi-arm multi-stage trials can improve the efficiency of finding effective treatments for stroke: a case study

**DOI:** 10.1186/s12872-018-0956-4

**Published:** 2018-11-27

**Authors:** Thomas Jaki, James M. S. Wason

**Affiliations:** 1 Department of Mathematics and Statistics, Lancaster University, Bailrigg, UK; 2 MRC Biostatistics Unit, Cambridge, UK; 3 Institute of Health and Society, Newcastle University, Baddiley-Clark Building, Claremont Road, Newcastle upon Tyne, UK

**Keywords:** Adaptive design, Clinical trial design, Multi-arm trials, Multi-arm multi-stage trials

## Abstract

**Background:**

Many recent Stroke trials fail to show a beneficial effect of the intervention late in the development. Currently a large number of new treatment options are being developed. Multi-arm multi-stage (MAMS) designs offer one potential strategy to avoid lengthy studies of treatments without beneficial effects while at the same time allowing evaluation of several novel treatments.

In this paper we provide a review of what MAMS designs are and argue that they are of particular value for Stroke trials. We illustrate this benefit through a case study based on previous published trials of endovascular treatment for acute ischemic stroke.

We show in this case study that MAMS trials provide additional power for the same sample size compared to alternative trial designs. This level of additional power depends on the recruitment length of the trial, with most efficiency gained when recruitment is relatively slow. We conclude with a discussion of additional considerations required when starting a MAMS trial.

**Conclusion:**

MAMS trial designs are potentially very useful for stroke trials due to their improved statistical power compared to the traditional approach.

## Background

It is well recognised that drug development is costly and time consuming [1] yet in recent years about half of Phase III trials and 80% of Phase II studies undertaken have been unsuccessful [2, 3]. While it is undesirable for every trial to conclude superiority of the experimental treatment – this would raise the question why such studies are done at all – it is widely agreed that these figures are unacceptably high. The situation in stroke is no better with several recent studies failing to show superiority of the experimental treatment [4–6]. In addition to the cost, many patients have been exposed to ineffective, possibly even harmful, treatments.

There are 19 distinct treatments for stroke currently under development [7] – about half undergoing Phase II studies. Accounting for different doses of the same treatment and combinations of treatments, the number of potential experimental treatment arms in stroke to be evaluated in trials in the next few years is huge. Evaluating all in traditional randomized controlled Phase III studies will lead to competition to recruit patients to each of the studies. A large number of patients will be allocated to control treatments, as each trial requires a separate control group. Moreover if past trends continue, many patients will be exposed to treatments that will ultimately be found to be ineffective. To address these issues, alternative designs need to be considered.

In this work we will discuss the potential utility of combining multiple experimental arms into a single multi-arm trial for improving evaluating treatments in stroke. We also consider going further and considering an adaptive approach called a multi-arm multi-stage (MAMS) design, which allows elimination of ineffective treatments while reducing the number of patients allocated to a control treatment.

We first consider these designs and their advantages in more detail. We then argue why they are particularly suitable for stroke trials. Next, we consider a case study that illustrates the potential advantages using an example of two randomised trials in endovascular treatment for acute ischemic stroke. We end the paper with discussion of benefits and limitations of multi-arm and MAMS designs.

## Main text

### Multi-arm trials

A multi-arm trial compares several different experimental treatments against a common control group within a single study. An immediate desirable consequence of this set-up is that only a single control group is required, reducing the number of patients on the control treatment compared to separate two-arm evaluations. The reduction in sample size for a trial with 3 experimental arms versus conducting 3 separate two-arm trials is around 15%. Additionally patients are more likely to be randomized to an experimental treatment – a feature that helps recruiting patients [8, 9]. Multi-arm designs also offer the unique opportunity for a fair head-to-head comparison of experimental treatments within the same study. This is due to the same patient population being studied, all patients following the same protocol and use of the same comparator group.

### Multi-arm multi-stage trials

Similarly to group-sequential designs for two arm studies [10], efficiency can be gained by the inclusion of interim analyses. At each interim analysis, test statistics based on all patients assessed up to that point are calculated to compare the effect of each (remaining) experimental treatment to control. These test statistics are used to select which treatment(s) should be continued and which should be stopped. An arm is stopped either because the evidence so far suggests that the treatment is unlikely to be superior to control (known as lack-of-benefit, or futility stopping) or because the accumulated evidence is already sufficient to claim superiority of that treatment arm (known as efficacy stopping). For the control arm and every remaining experimental arm, further patients are recruited until a decision has been reached or until a maximum number of analyses is reached.

**Fig. 1:**
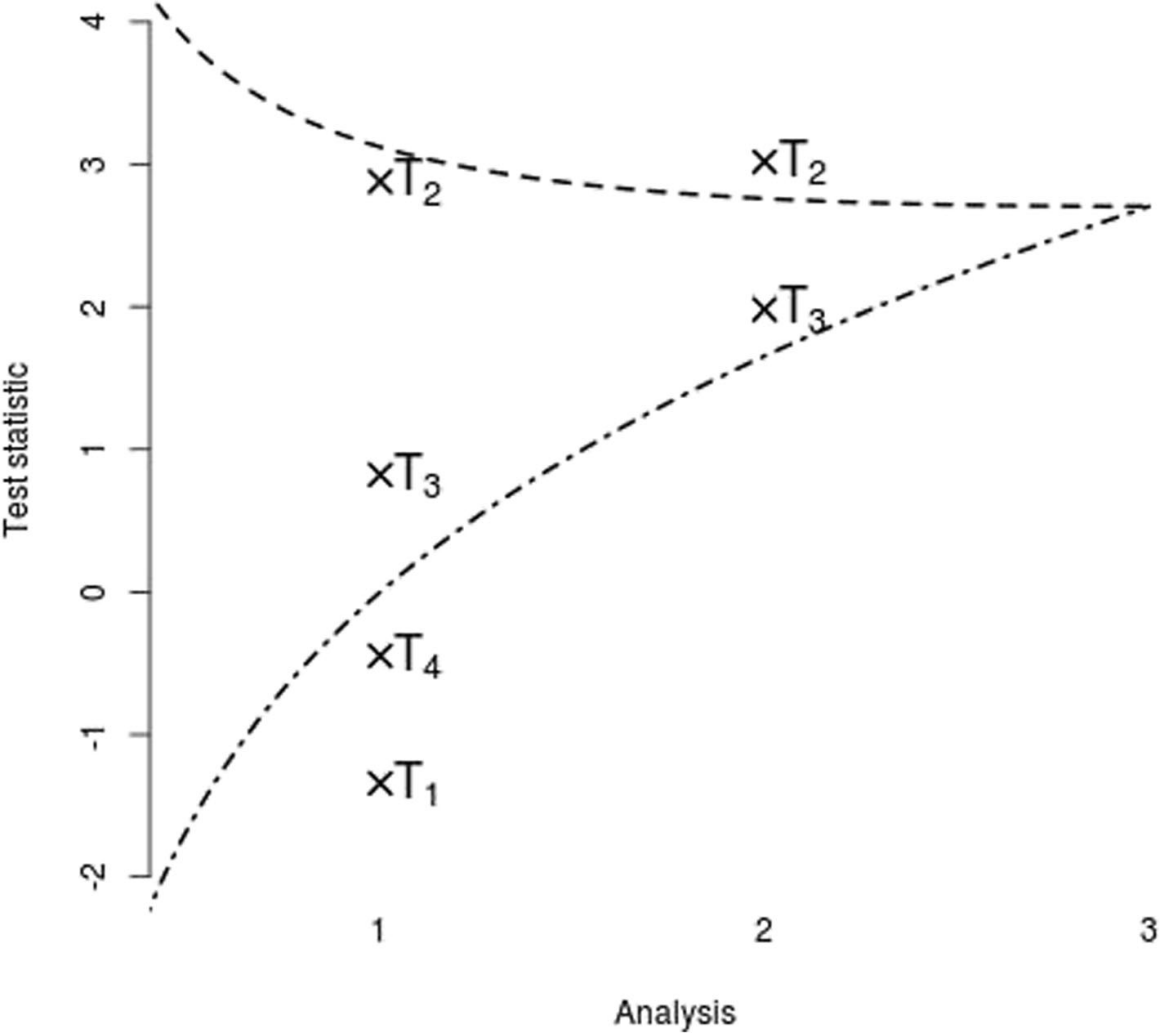
Illustration of a multi-arm multi-stage design. Crosses represent the test statistic at each analysis for each of the experimental arms against control. The upper dashed line represents the efficacy boundary (with a treatment being recommended as superior to control if the test statistic is above this), and the lower dash-dotted line represents the futility boundary (with the treatment being stopped early for lack of benefit if the test statistic is below this)

Figure 1 illustrates such a multi-arm multi-stage (MAMS) design. In this example four experimental treatments and three analysis time points are used. At the first interim analysis, the test statistics for treatment 1 and 4 are below a pre-specified lower bound implying that they are unlikely to be superior to control. As a consequence no further patients are randomized to these treatments. Neither of the test statistics for treatment 2 and 3 exceeds the upper bound of the design at the first analysis so further patients are randomized to those treatments and control. At the second analysis, the test statistic for treatment 2 exceeds the upper boundary so that superiority for this treatment over control can be concluded. In this example no further patients are randomized as a successful treatment has already been found. Alternatively, one could continue with the last remaining treatment and control until a definitive decision for this treatment has also been reached.

Different ideas have been proposed for how to select which experimental treatments should continue at the interim analyses. The example above selects all treatments that are deemed sufficiently promising [11, 12] while alternatively only the best or the best few could also be chosen [12]. Common to all these designs is that by allowing for treatment selection the number of patients recruited in the trial is typically markedly smaller than without treatment selection at an interim analysis.

### Practical considerations when conducting multi-arm multi-stage designs

As argued above, a multi-arm design will be more efficient than separate two-arm studies. However, a few points need to be noted. Firstly, despite the sample size often being smaller than for a study without interim analysis, there is a small chance that the sample size is increased as well. This is due to the need to account for possible wrong decisions at the interim analyses. Secondly, (notable) reductions in patient numbers are only possible if the endpoint utilized for treatment selection (typically the primary endpoint or some short-term surrogate) is observed quickly relative to the recruitment rate. The reason for this is that, in an extreme scenario, all patients could already be recruited by the time the information from assessed patients is available for making the treatment selection decision. Thirdly, since all of the experimental treatments start at an equal footing, none of them should have strong evidence of superiority to the others at the onset of the study. In the case where there is a treatment that is highly likely to be the best treatment, the most efficient approach would be to just test that treatment against control. Finally, the organization of interim analyses must be efficient, with data monitoring and statistical analysis done to tight deadlines [13]. Additional resources are required to conduct and additional effort to maintain blinding. However, these issues are also present in traditional RCTs as data monitoring committees are regularly provided with unblinded trial data.

In addition to the general considerations above – most of which are also relevant for traditional two-armed studies with interim analyses, some additional administrative and operational aspects need to be considered which mean that the time to set up a MAMS study is typically longer than for a traditional study, although substantially less than the time to set up multiple separate trials. Firstly, different trials often are initiated by different centres, have different inclusion and exclusion criteria, and may use different primary and secondary endpoints. All of these must be standardised for a multi-arm trial which may require negotiations and compromises between investigators.

Secondly, it is more difficult to explain MAMS trials to patients. Providing informed consent requires that patients are aware of all the possibilities. The STAMPEDE trial, for example, addressed this through use of a two-part patient information sheet [14]: summary information on all arms is provided before randomisation; detailed information of the allocated therapy is provided after randomisation. The more detailed information can be requested on all arms prior to randomisation.

Planning and ensuring treatment supply poses the third challenge. Due to the fact that arms can be stopped, the maximum drug supply for each arm is uncertain. While the same can be said for group-sequential designs this issue is more pronounced in MAMS studies due to the use of multiple arms. This issue is further exaggerated when multiple centres and countries are taking part in the study. Consequently the use of advanced prediction approaches for multi-centre trials is paramount [15].

A fourth challenge is to ensure that no bias in the evaluation is introduced by an imbalance in the allocation of treatments across centres and regions. It is therefore important to stratify randomization by centre or region. Doing so also simplifies prediction of the drug supply to the different centres as within centre imbalances are reduced.

It is our belief that more complex administration and operation is vastly outweighed by the improved efficiency and reduced exposure of patients to potentially harmful treatments. These administration and operation hurdles have successfully been overcome in a variety of other therapeutic areas [16–20]. Even within Stroke, the ASTIN study has been undertaken [21]. Thus, we believe that some of the objections often raised are based on misconceptions. For example, concerns about a much more elaborate ethical approval process rarely hold true. As a MAMS design is a single study under a single protocol, the only difference in obtaining approval is that now several treatments need to be deemed worthy of experimentation. Concerns about public funders being unwilling to fund such designs have also been proven false in other areas [17, 22, 23].

### Why MAMS trials are suitable for stroke

So far our description of MAMS designs has been generic and made little reference to the specifics of stroke trials. It is our belief that trials in stroke are particularly suited for MAMS. The reasons include:

1. Numerous treatments/regimens are currently in the early development stages and hence soon available for large scale testing [7].
2. Trials in stroke tend to be large resource intensive studies. Eliminating ineffective treatments prevent delays in evaluating alternative treatments. Additionally the savings in resources from dropping an ineffective arm are large.
3. The primary outcome measure, often the modified Rankin scale [24] or the Barthel index [25], are usually measured 90 days after treatment initiation and hence quickly observed.
4. Current failure rates are high so it can be expected that many treatments can be eliminated from the study quickly.
5. Information about the relative merits of different treatment options is limited due to success in early studies being a poor predictors of success in late stage trials [26].

In the next section we will illustrate, based on real studies, the potential utility of multi-arm multi-stage designs in stroke.

### Case study

We consider two completed randomised controlled trials of endovascular treatment for acute ischemic stroke with similar inclusion criteria (Ciccone et al. [5], Broderick et al. [6]) that both used intravenous t-PA therapy as the control. Ciccone et al. [5] used endovascular therapy alone as the experimental treatment while intravenous t-PA with endovascular therapy was used in Broderick et al. [6] Both trials used a dichotomisation of the modified Rankin scale at 3 months as the primary outcome. A score of 0 or 1 was defined as a success in Ciccone et al. while a score of 0–2 was classed as a success in Broderick et al. Neither trial found a significant difference between the experimental therapy and the control arm. Table 1 shows the percentage of patients for each of the modified Rankin categories in each treatment arm. Irrespective of the definition of success used, both experimental arms were slightly worse than intravenous t-PA.

**Table 1:** Proportion of patients in each category of the modified Rankin score from Ciccone et al. [5] and Broderick et al. [6]

Modified Rankin score	Intravenous t-PA (*n* = 395)	Endovascular only (*n* = 181)	Endovascular and intravenous t-PA (*n* = 415)
0	11.9%	12.2%	12.8%
1	18.7%	18.2%	16.6%
2	12.4%	11.5%	13.3%
3	15.9%	20.4%	17.1%
4	17.2%	17.7%	15.4%
5	7.1%	5.5%	4.8%
6	16.7%	14.3%	20.0%
Odds ratio (success = 0 or 1)		0.991	0.944
Odds ratio (success = 0–2)		0.956	0.988

Subsequently we will compare several design possibilities in terms of the power and sample size required. These are: 1) two separate trials, each testing one of the experimental treatments against control; 2) a three-arm MAMS design; 3) two separate group-sequential trials, each testing one of the experimental treatments against control; 4) a multi-arm trial but no possibility of early stopping. In reality, the sample size used in each trial was not the same, so we simplify comparisons by considering equal numbers of patients recruited per arm in the multi-arm and MAMS trial, and equal numbers of patients in the separate trial and separate group-sequential trials setting. The total number of patients assessed in the two trials was 991. To allow fair comparisons, we set the number of patients per arm in each case so that the maximum sample size was close to this value. The two separate trials each have a sample size of 248 per arm (992 in total), the MAMS trial has 165 patients per arm per stage (a maximum of 990), the two separate group-sequential designs each have 124 patients per arm per stage (maximum 992) in total, and the multi-arm trial recruits 330 patients per arm (total 990).

For both the MAMS and separate group-sequential trials, we include a single interim analysis that eliminates treatments for futility. Triangular stopping boundaries [27] (Fig. 1) are used, due to its good properties [28]. For the MAMS trial, the futility test statistic threshold is 0.662 and the final critical value is 1.866. In terms of *p*-values, this is equivalent to a futility threshold of 0.254 (i.e. if the *p*-value for a comparison is above 0.254 at the interim the corresponding experimental arm is dropped) and a final critical value of 0.031 (i.e. superiority can be concluded if the final *p*-value is below 0.031). Each separate group-sequential trial has a futility test statistic threshold of 0.678 and a final critical value of 1.917 (in terms of *p*-values, 0.249 and 0.028 respectively). These values and the critical values used in the other two designs were chosen to limit the maximum chance of making a type I error to 5%.

We consider two scenarios: 1) the three treatments (two experimental and control) have the same treatment effect as found in the real trials (Table 1, with success defined as a 0 or 1); 2) a hypothetical scenario where intraveneous t-PA and endovascular therapy alone both have the success probabilities of 0.306, and intraveneous t-PA combined with endovascualar therapy has a success probability of 0.406. The statistical characteristics of the MAMS designs and the two separate trials are calculated by using a normal approximation for the log-odds ratio and applying methods from Magirr et al. [11] These results are summarised in Table 2. For the first scenario, the probability of recommending a truly ineffective treatment and the expected sample size is given. For the second scenario, the probability of recommending the truly effective treatment and the expected sample size is given.

**Table 2:** Properties of MAMS design and running two separate trials

Design	Treatments ineffective		One treatment effective	
	Total type-I error rate	Expected sample size	Power to recommend effective treatment	Expected sample size
MAMS, futility only, two-stage	0.033	623	0.761	834
Separate trials	0.034	992	0.644	992
Separate trials, group-sequential	0.036	608	0.625	764
Multi-arm, no interim analyses	0.036	990	0.782	990

As described further in the manuscript, the ‘Treatments ineffective’ scenario uses a success probability of 0.304 for the control treatment, 0.302 for the first experimental treatment and 0.294 for the last arm. The ‘one treatment effective’ scenario uses 0.404 for one experimental treatment while the other two arms use 0.304. Statistical properties are found by approximating the log-odds ratio as normally distributed

**Fig. 2:**
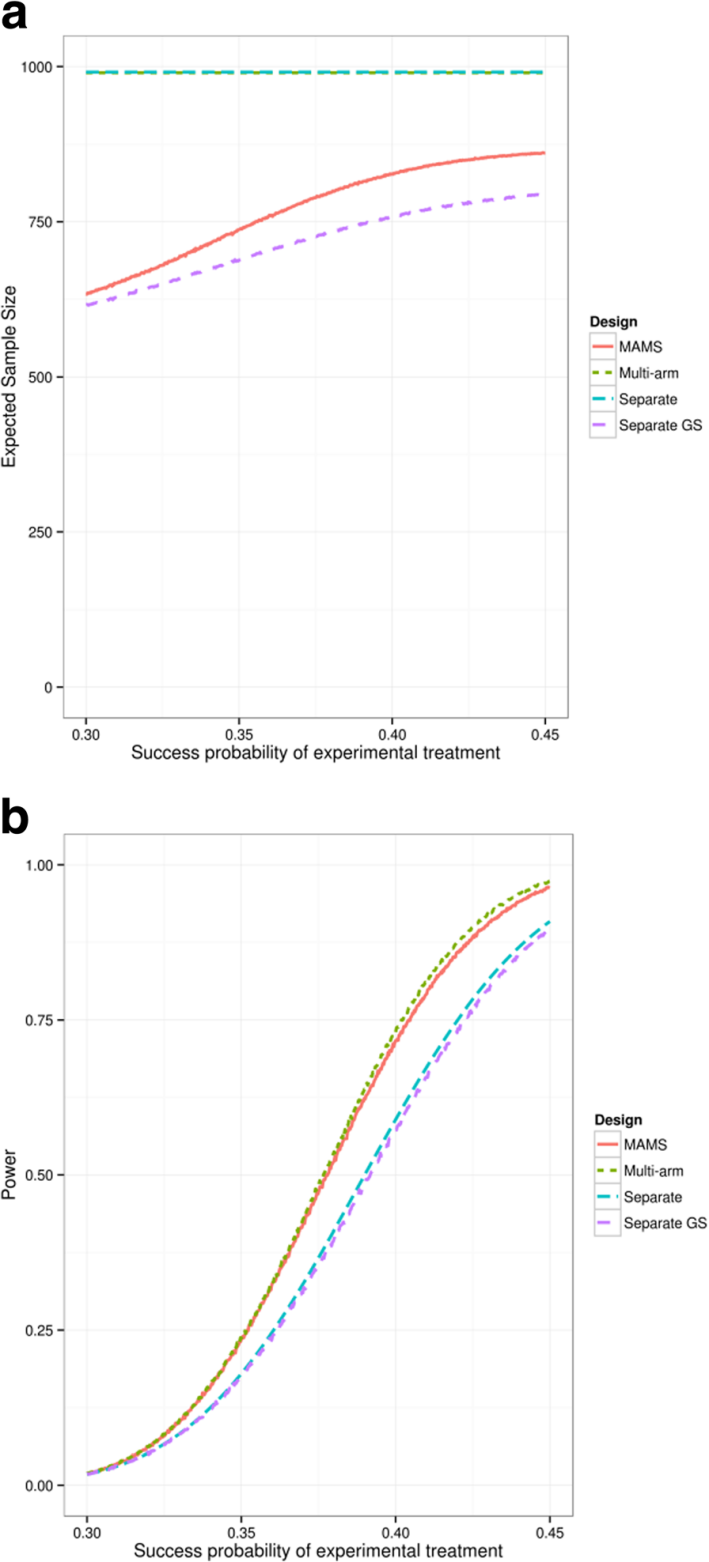
Plots of **a**) power and **b**) expected sample size of separate trials and MAMS trial as the probability of success of one experimental arm changes. Probability of success for the other arms is 0.309. MAMS: multi-arm multi-stage; GS: group-sequential

Table 2 shows that the MAMS design has a high power and a low expected sample size. It has a considerably lower expected sample size than separate trials and the multi-arm trial, but has a considerably higher power than separate trials and separate group-sequential trials. This indicates the MAMS design is likely to drop ineffective treatments. The type I error rate for all approaches is similar. Figure 2 shows the expected sample size and power of all four designs as the effectiveness of the second experimental arm varies. The power of the MAMS trial is close to the multi-arm trial (which has the highest power) while the expected sample size is close to that of the separate group-sequential trials design, especially when the treatment effect is low. Although separate group-sequential trials have a lower expected sample size than the MAMS study, this reduction comes at the cost of substantially reduced power (which corresponds to a 37% drop in sample size).

**Fig. 3:**
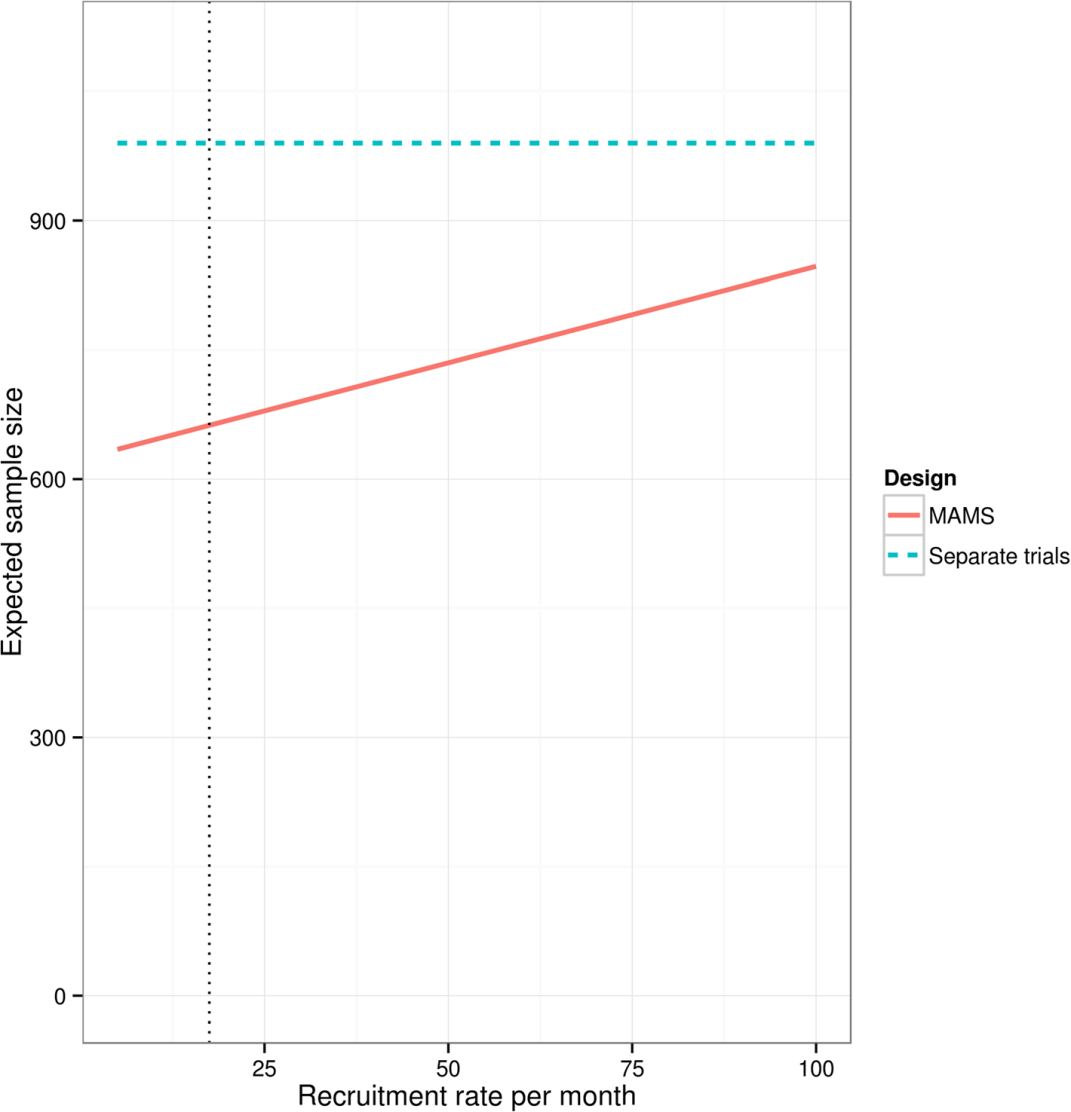
Expected sample size of MAMS design as monthly recruitment rate changes. Constant recruitment is assumed and a 90 day delay is assumed between recruitment and observing the primary endpoint. Vertical dotted line represents recruitment rate of trials described in Ciccone et al. and Broderick et al. combined

The results in Table 2 and Fig. 2 ignore the fact that there is delay between recruiting an individual and assessing the modified Rankin score. This delay causes a loss of efficiency in trial designs that use interim analyses which depends on the length of the delay and the recruitment rate. Hence using a quickly observed outcome will ensure that this loss is kept small. Figure 3 shows the expected sample size of the MAMS design, for a 90-day delay, as the recruitment rate of the trial increases. The efficiency, in terms of expected sample size, of the MAMS design goes down as the recruitment rate increases. The recruitment of the two previous two trials [5, 6] combined is around 17.5 patients per month. For this rate, there is still a substantial advantage to using a MAMS design, primarily because of the short assessment delay in stroke trials. We do not recommend purposely slowing recruitment, as this would increase the trial costs in different ways. Instead we recommend considering the likely recruitment rate and whether the MAMS design would provide an advantage in that case.

## Discussion

### Benefits of MAMS

In this manuscript we illustrate, based on real trial examples, that multi-arm multi-stage (MAMS) designs allow efficient evaluation of multiple treatments as ineffective treatments are quickly eliminated while the number of patients on control is kept small. Although our evaluation is focussed on evaluating 2 experimental arms simultaneously, gains in efficiency are even larger as the number of experimental arms increases. Another point to note here is that the different arms could also be different doses of the same treatment or combinations of treatments.

### Limitations of MAMS

There are clear efficiency reasons to use a MAMS design, yet there are a number of practical challenges that need to be considered when embarking on their use. In order to be able to utilize these different arms in a MAMS design, treatments must be available for testing at the same time making it crucial to coordinate sponsors, investigators and ethical and regulatory approvals. Additionally the consent procedures and information to patients are more complex than for standard two-arm studies as they need to account for all potential treatments in the study.

An interesting question arising in MAMS trials is whether or not it is important to control the total chance of making a type I error. In the case of running separate trials, it would not normally be required to adjust the significance level used in one trial because of the existence of another trial in the same condition. However arguably it is important to control a trial’s chance of recommending an ineffective treatment, which would imply it is necessary to control the chance of making any type I error. Some other papers [29, 30] have considered this debate in more detail, and we refer the interested reader to them.

Other types of MAMS designs have also been proposed, including using adaptive randomisation [31] and drop-the-losers [32]. Arguably it is a limitation that there is an array of design options available as it makes it confusing which one should be used in practice. Clearly careful consideration at the design stage of a MAMS trial is required.

## Conclusion

Despite the practical and statistical challenges, we believe that the benefits derived from MAMS designs clearly outweigh running multiple two-arm studies, so that they should be considered regularly for stroke trials. As these designs become more regularly used the expertise gained in setting up such studies will help to streamline their implementation even further.

## Abbreviation

MAMS: multi-arm multi-stage
